# Echocardiography Finds an Unusual Cause of Atrial Fibrillation

**DOI:** 10.1016/j.case.2026.02.002

**Published:** 2026-03-26

**Authors:** Tomoya Hasegawa, Junya Tanabe, Hiroshi Kawahara, Taiji Okada, Nobuhide Watanabe, Akihiro Endo, Kazuaki Tanabe

**Affiliations:** Division of Cardiology, Shimane University Faculty of Medicine, Izumo, Japan

**Keywords:** Venous extension, Cardiac invasion, Atrial fibrillation, Small-cell lung cancer, Echocardiography

## Abstract

• Venous extension into the LA is a known complication of SCLC.
• This complication may be associated with the onset of AF.
• Multimodal imaging is required to assess tumor extension into the heart.
• This case raises a possible mechanistic link between cancer extension into the LA and AF.

## Graphical abstract

**Figure:**
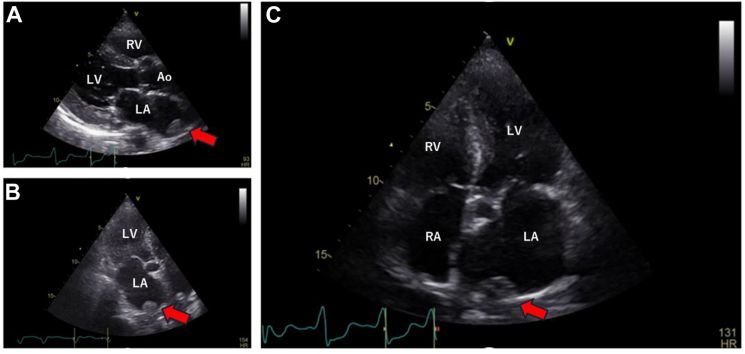

## Introduction

Cardiac metastasis is tumor dissemination to the heart via hematogenous or lymphatic routes from distant organs, whereas cardiac involvement from adjacent structures, such as lung cancer or mediastinal tumors, occurs through direct invasion into the myocardium or venous extension into the cardiac chambers. Compared to metastasis, these forms of cardiac involvement can cause more significant alterations in cardiovascular structure and hemodynamics, potentially leading to rapid clinical deterioration—such as shock—due to cardiac tamponade or vascular obstruction. Furthermore, while cardiac metastasis from lung cancer is generally managed with chemotherapy in patients with stable systemic conditions, some cases of localized cardiac extension have been reported to achieve favorable outcomes through surgical intervention.^1^ Therefore, distinguishing the precise mechanism of cardiovascular involvement is important. With advances in imaging modalities and prolonged survival of patients with cancer, the detection of cardiac extension is expected to increase. Notably, since tumor extension into the heart often remains asymptomatic until it reaches an advanced stage, the onset of refractory atrial fibrillation (AF) serves as a critical sentinel manifestation for the early detection of venous extension into the left atrium (LA). While AF is common in older adults, it serves as a crucial primary indicator of cardiac tumor extension into the LA in lung cancer.^2^ Here, we report a case of small-cell lung cancer (SCLC) extending into the LA via the pulmonary vein (PV), detected by transthoracic echocardiography (TTE) following the onset of refractory AF and subsequent acute heart failure.

## Case Presentation

**Figure 1:**
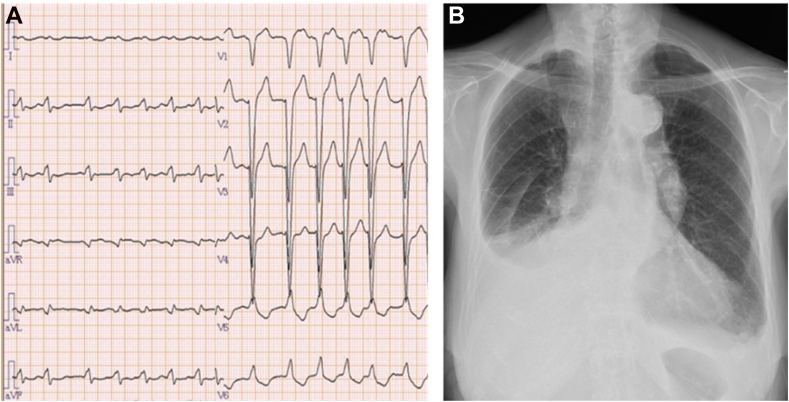
Twelve-lead electrocardiogram on admission demonstrates AF with a heart rate of 129 beats/min and complete left bundle branch block pattern **(A)**. Chest x-ray on admission, posterior-anterior display, demonstrates a cardiothoracic ratio of 58% and right-sided pleural effusion and age-related vascular and bony changes **(B)**.

An 82-year-old woman with a history of SCLC in the right lower lobe (T4N0M0) treated with chemoradiotherapy 2 years earlier presented with palpitations during routine follow-up. On admission, the vital signs were as follows: blood pressure, 91/65 mm Hg; irregular pulse rate, 120 beats/min; and oxygen saturation, 95% on room air. Physical examination revealed mild pitting edema in both lower extremities. A 12-lead electrocardiogram revealed AF with a ventricular rate of 129 beats/min and complete left bundle branch block. Chest radiography revealed a cardiothoracic ratio of 58% and right-sided pleural effusion (Figure 1). Based on these findings, the patient was diagnosed with acute decompensated heart failure secondary to AF with rapid ventricular response and admitted to the hospital.

**Figure 2:**
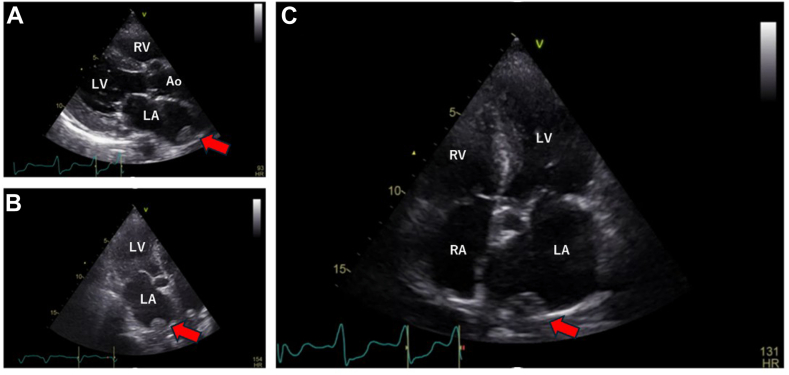
Two-dimensional TTE, parasternal long-axis **(A)**, apical 3-chamber **(B)**, and apical 4-chamber **(C)** systolic views, demonstrates the dilated LV and LA and a large, nonmobile, hyperechoic mass *(arrows)* protruding into the posterior wall of the LA from the orifice of the RLPV. *Ao*, Aorta; *LV*, left ventricle; *RA*, right atrium; *RV*, right ventricle.

**Figure 3:**
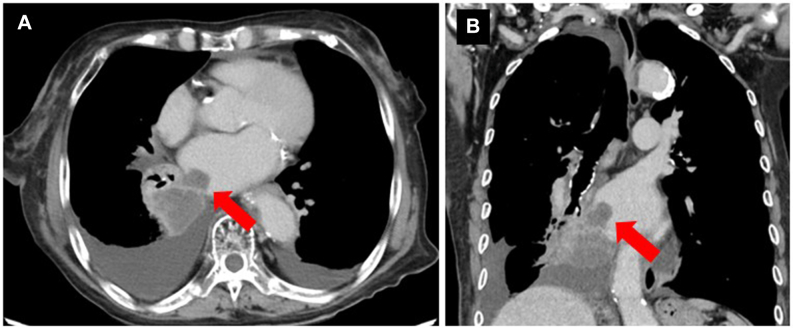
Cardiac CT, axial **(A)** and oblique coronal **(B)** views obtained during the portal venous phase, demonstrates the pleural effusions and the large, hypodense SCLC tumor mass with extension from the RLPV into the LA *(arrows)*.

Transthoracic echocardiography revealed a left ventricular ejection fraction (LVEF) of 24%, measured using the modified Simpson's method. The left atrial diameter was 27 mm, indicating no dilation, and the estimated central venous pressure was elevated at 8 mm Hg. A well-defined round mass measuring 18 × 11 mm was identified protruding into the LA from the right lower PV (RLPV). The mass was isoechoic to the myocardium and exhibited a slightly heterogeneous internal texture without mobility (Figure 2, Videos 1-3). Cardiac computed tomography (CCT) performed during the portal venous phase revealed bilateral pleural effusions and a well-defined hypodense mass with no contrast enhancement that was continuous with the SCLC in the right lower lobe and had extended into the LA via the RLPV. On CCT, the portion of the mass protruding into the LA measured 18 × 17 × 14 mm. The lumen of the RLPV was obliterated; however, no extension into other PVs was observed. No thrombus was identified in the visualized portion of the left atrial appendage, and no other intracardiac masses were detected (Figure 3).

Hemodynamics gradually stabilized following rate control with beta-blockade and diuretic therapy. Spontaneous conversion to sinus rhythm occurred on day 5 of hospitalization; however, AF recurred within 2 days and persisted thereafter despite intravenous antiarrhythmic therapy. On day 15, thoracentesis was performed for the right pleural effusion, but the cytological results were negative for malignant cells. The patient declined further diagnostic evaluation and cancer-directed therapy and opted for palliative care. During hospitalization, no progression of the pericardial effusion or thromboembolic events was observed. However, due to the deterioration of the general condition of the patient, discharge to home was considered difficult, and the patient was transferred to a rehabilitation hospital on day 34.

**Figure 4:**
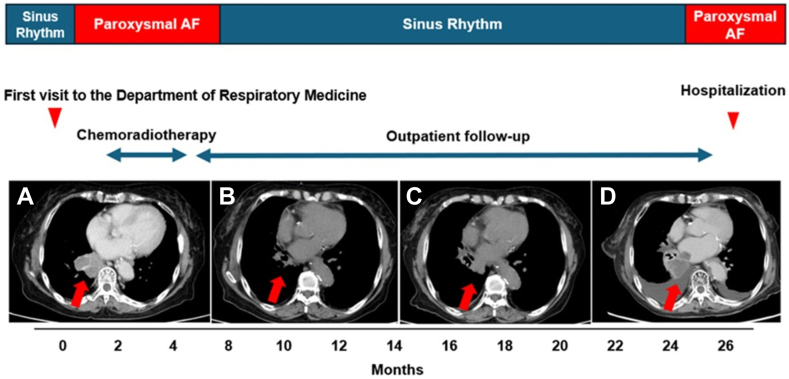
Clinical course timeline illustrates the association of AF episodes and the visualized left lower lobe SCLC tumor progression *(arrows)*. Contrast-enhanced CT at the initial diagnosis 2 years prior to admission **(A)** demonstrates tumor extension into the RLPV; noncontrast CT after chemoradiotherapy, demonstrates significant tumor shrinkage **(B)** with mild tumor regrowth at 6 months **(C)**; CCT during this case presentation demonstrates further tumor growth with intracardiac protrusion **(D)**.

**Figure 5:**
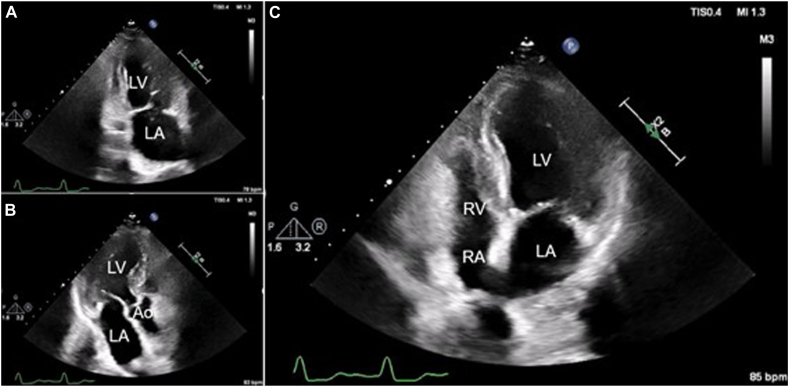
Two-dimensional TTE, apical 2-chamber **(A)**, apical 3-chamber **(B)**, and apical 4-chamber **(C)** systolic views performed 2 years earlier, demonstrates normal left ventricular and left atrial size without visualized mass. *LV*, Left ventricle; *RA*, right atrium; *RV*, right ventricle.

The clinical course of AF episodes and tumor progression prior to admission is shown in Figure 4. Two years earlier, the patient presented to our respiratory medicine department and was diagnosed with SCLC in the right lower lobe adjacent to the heart. The patient was hospitalized for approximately 2 months to undergo chemoradiotherapy, consisting of carboplatin and etoposide combined with radical radiotherapy (60 Gy in 30 fractions). During this hospitalization, paroxysmal AF was documented, which prompted a referral to the cardiology department. At that time, TTE performed during sinus rhythm revealed an LVEF of 49%, measured using the modified Simpson's method, with paradoxical septal motion due to complete left bundle branch block. The left atrial diameter was 31 mm, and the left atrial volume index was 46 mL/m^2^. No obvious mass was observed within the LA (Figure 5, Videos 4-6). Contrast-enhanced computed tomography (CT) at that time suggested tumor extension into the RLPV, although there was no evidence of protrusion into the LA. After successful chemoradiotherapy and tumor shrinkage, the patient remained free of palpitations and AF for >1 year. However, AF recurrence coincided with tumor regrowth adjacent to the heart. During outpatient follow-up, tumor markers remained consistently negative, and progressive pleural effusion was noted. Thoracentesis was performed twice, and both yielded negative cytological results for malignant cells.

## Discussion

Among primary tumors that spread to the heart, lung cancer is the most common, accounting for approximately 40%, followed by lymphomas, leukemias, and breast cancers.^3^ Lung cancer frequently extends to the heart through direct invasion, and left atrial invasion via the PV is well recognized.^4^ A standard therapeutic strategy for venous extension into the LA in lung cancer has not yet been established, and further research is required. Renal cell carcinoma often progresses to the right atrium through the inferior vena cava.^5^ Hematogenous metastases are most common in melanoma and sarcomas, whereas lymphatic spread is typical of lung and breast cancers.^6^

Clinical manifestations such as dyspnea, palpitations, arrhythmias, chest pain, or pericardial effusion are typically nonspecific, which makes diagnosis challenging. Patients with malignancies have shown a higher prevalence and incidence of AF, which may be triggered by tumor infiltration or mechanical compression of the heart as well as by inflammatory mechanisms.^7^ Interestingly, recent retrospective cohort studies have suggested a bidirectional relationship between AF and cancer.^8^^,^^9^ When tumors extend into the atrium or PV, the proposed mechanisms for AF include enhanced micro-reentry activity, localized mechanical irritation of the atrial wall, and autonomic nervous system activation.^10^

Generally, it has been suggested that chemotherapy may contribute to an increased risk of AF during or following treatment, possibly by causing structural and/or electrical remodeling, inducing and maintaining inflammation and/or causing cardiac damage and cellular apoptosis.^11^ However, compared to agents with established cardiotoxicity such as anthracyclines, we did not strongly suspect the etoposide and carboplatin used in our patient to be direct triggers for AF. Instead, prior radiotherapy appears to be a more significant independent factor.^12^

In the present case, paroxysmal AF initially occurred during chemoradiotherapy, likely triggered by the tumor extending into the PV-LA junction, which is a well-known site of arrhythmogenicity. The AF subsided following tumor shrinkage and recurred with regrowth of the tumor adjacent to the LA. This temporal correlation suggests that left atrial involvement through the PV acted as the primary trigger for AF recurrence. Although the potential contribution of prior radiotherapy cannot be entirely excluded, the fact that AF subsided and recurred in tandem with tumor size changes underscores the clinical significance of cardiac extension. Tumor-related AF is often refractory to treatment; recurrence of AF in cancer populations with higher risk of cardiac extension should raise suspicion and warrant earlier imaging evaluation. Therefore, the appearance of refractory AF in patients with cancer should prompt consideration of cardiac extension. Previous reports of refractory AF caused by atrial involvement of SCLC support this observation.^13^

The pathophysiology of heart failure in this case warrants careful consideration. The patient's baseline LVEF was 49% prior to chemoradiotherapy but declined to 24% upon the current admission. Although certain cancer therapies are known for cardiotoxicity, the chemotherapy used—carboplatin and etoposide—is not typically associated with direct, severe cardiotoxicity, unlike agents such as anthracyclines. Furthermore, the radiation dose was carefully planned to avoid direct ventricular exposure. Given the absence of cardiotoxic agents and the temporal correlation between tumor regrowth and refractory AF, we suspect that the primary driver of heart failure was tachycardia-induced cardiomyopathy rather than direct treatment-related toxicity.

Transthoracic echocardiography is the most common modality for the diagnosis of cardiac involvement because it is noninvasive, readily accessible, and useful not only for detecting intracardiac masses and pericardial effusion but also for providing real-time assessment of mass mobility and global cardiac function. However, its diagnostic utility may be limited by a restricted acoustic window and a narrow field of view for posterior structures, such as the PV. Furthermore, TTE lacks advanced tissue characterization capabilities, making it difficult to definitively differentiate tumors from thrombi or artifacts. Thrombi typically appear more homogeneous and are usually located in the left atrial appendage or regions of blood stasis, whereas tumors often exhibit heterogeneous echogenicity due to internal necrosis or vascularity. In the present case, the location of the mass at the PV orifice and its heterogeneous internal texture raised suspicion for a tumor; nevertheless, differentiation from a thrombus remained challenging based on TTE findings alone. Compared to TTE, transesophageal echocardiography (TEE) offers superior spatial resolution and a closer acoustic window for evaluating venous extension into the LA without lung interference. However, TEE is a semi-invasive procedure and carries potential risks, including aspiration or hemodynamic instability during sedation. In our patient, TEE was not performed because of unstable hemodynamics during the acute phase. After stabilization, further invasive diagnostic procedures were not pursued, as the patient and family did not wish to undergo aggressive investigations. Although not utilized in the present case, ultrasound-enhancing agents (UEAs) can offer substantial diagnostic value, particularly for masses located in the far-field regions such as the LA. Ultrasound-enhancing agents improve endocardial border delineation and, more importantly, allow assessment of intralesional perfusion. Malignant tumors typically demonstrate contrast enhancement owing to neovascularization, whereas thrombi are avascular and therefore lack enhancement.^14^ Consequently, UEAs can enhance the diagnostic performance of TTE in differentiating malignant extension from thrombus, especially when more invasive modalities such as TEE are not feasible.

Computed tomography allows comprehensive evaluation of the whole body with excellent spatial resolution, enabling the simultaneous assessment of the cardiac anatomy, adjacent structures, and distant metastases. In the evaluation of cardiac masses, CCT is instrumental in detecting neovascularization. Typically, malignant invasions present as hypodense or isodense areas relative to the enhanced myocardium, which serves as a crucial clue to differentiate vascularized tumors from thrombi that characteristically lack enhancement due to their avascular nature. In this case, the mass demonstrated poor contrast enhancement and appeared hypodense on CCT. This appearance was attributed to the fact that the CCT images were acquired during the portal venous phase, without the opportunity to demonstrate gradual accumulation of contrast medium within the tumor, which might have been shown with a delayed acquisition protocol. Alternatively, it might reflect the hypovascular nature or extensive internal necrosis of the tumor itself. While a delayed-phase protocol might have provided further tissue characterization, the direct anatomical continuity observed between the primary lung lesion and the intracardiac mass through the PV provided evidence of tumor extension.

^18^F-fluorodeoxyglucose positron emission tomography/CT (FDG PET/CT) is another noninvasive modality for differentiating malignant masses from thrombi. Malignant tumors, including SCLC, typically exhibit high FDG uptake due to increased glucose metabolism, whereas thrombi generally do not show significant uptake. It should be noted, however, detecting cardiac extension with FDG PET/CT can be challenging because the intense physiological FDG uptake by the normal myocardium can mask tumor-related signals. Although specific protocols, such as prolonged fasting or high-fat, low-carbohydrate diets, are required to suppress normal myocardial glucose metabolism, the metabolic information provided by PET/CT is particularly valuable in cases where contrast-enhanced imaging is limited by impaired renal function or when internal enhancement patterns are equivocal.

Cardiovascular magnetic resonance (CMR) provides superior tissue characterization and can differentiate between tumors and thrombi, while also delineating the extent of cardiac involvement. On T2-weighted imaging, malignant tumors typically show high signal intensity due to edema, whereas T1-weighted imaging and fat saturation sequences can help identify hemorrhage or fatty components. Notably, late gadolinium enhancement CMR offers higher contrast resolution than CCT, enabling a more detailed evaluation of internal enhancement patterns. In cases where a tumor exhibits poor contrast uptake on portal venous phase CCT, mimicking the appearance of a thrombus, late gadolinium enhancement CMR is often capable of detecting subtle, delayed contrast accumulation within the mass.^15^ This provides a significant advantage in distinguishing a malignant lesion from a truly avascular thrombus.

Malignant cardiac spread can be classified into pericardial, myocardial, and endocardial stages. Identifying the precise extent of tumor extension is critical for management because each stage results in distinct clinical manifestations and necessitates different therapeutic strategies. Pericardial involvement may present with cardiac tamponade requiring urgent drainage, whereas myocardial infiltration can lead to fatal arrhythmias or contractile dysfunction, and endocardial extension carries a high risk of systemic thromboembolism or valvular obstruction. Therefore, multimodal imaging plays a vital role in this differentiation. Transthoracic echocardiography is highly effective for the real-time assessment of pericardial effusion, such as detecting effusion that may progress to cardiac tamponade, while CMR provides superior tissue characterization for evaluating myocardial invasion. Cardiac CT and FDG PET/CT offer excellent spatial resolution for delineating endocardial extension and anatomical relationships with adjacent structures. Therefore, lung cancer with venous extension into the LA, as in the present case, represents a condition with limited treatment options, necessitating an accurate diagnosis and individualized treatment strategies.

## Conclusion

Cardiac invasion or metastasis should be considered in the differential diagnosis of patients with cancer who develop new-onset or treatment-refractory arrhythmias. Multimodal imaging plays a crucial role in establishing a diagnosis.

## Ethics Statement

The authors declare that the work described has been carried out in accordance with The Code of Ethics of the World Medical Association (Declaration of Helsinki) for experiments involving humans.

## Consent Statement

The authors declare that since this was a non-interventional, retrospective, observational study utilizing de-identified data, informed consent was not required from the patient under an IRB exemption status.

## Funding

The authors declare that this report did not receive any specific grant from funding agencies in the public, commercial, or not-for-profit sectors.

## Disclosure Statement

The authors reported no actual or potential conflicts of interest relative to this document.

## Supplementary Data

Video 1 Two-dimensional TTE, parasternal long-axis view, demonstrates AF, dilated left ventricle with reduced left ventricular systolic function and a large, nonmobile, hyperechoic mass protruding into the posterior wall of the LA from the orifice of the RLPV.

Video 2 Two-dimensional TTE, apical 3-chamber view, demonstrates AF with a rapid ventricular response, dilated LV with reduced left ventricular systolic function, and a dilated LA with a large, nonmobile, hyperechoic mass protruding into the posterior wall of the LA from the orifice of the RLPV.

Video 3 Two-dimensional TTE, apical 4-chamber view, demonstrates AF with a rapid ventricular response and a large, nonmobile, hyperechoic mass protruding into the posterior wall of the LA from the orifice of the RLPV.

Video 4 During the hospitalization for chemoradiotherapy 2 years prior, two-dimensional TTE, apical 2-chamber view performed 2 years earlier, demonstrates normal left ventricular size and function and a normal sized LA without visualized mass.

Video 5 During the hospitalization for chemoradiotherapy 2 years prior, two-dimensional TTE, apical 3-chamber view performed 2 years earlier, demonstrates normal left ventricular size and function, abnormal septal motion consistent with left bundle branch block, and a normal sized LA without visualized mass.

Video 6 Two-dimensional TTE, apical 4-chamber view performed 2 years earlier, demonstrates normal left ventricular size and function, abnormal septal motion consistent with left bundle branch block, and a normal sized LA without visualized mass.
